# Improving Adoptive T Cell Therapy: The Particular Role of T Cell Costimulation, Cytokines, and Post-Transfer Vaccination

**DOI:** 10.3389/fimmu.2016.00345

**Published:** 2016-09-06

**Authors:** Anke Redeker, Ramon Arens

**Affiliations:** ^1^Department of Immunohematology and Blood Transfusion, Leiden University Medical Center, Leiden, Netherlands

**Keywords:** adoptive cell therapy, T cells, costimulation, cytokines, vaccination

## Abstract

Adoptive cellular therapy (ACT) is a form of immunotherapy whereby antigen-specific T cells are isolated or engineered, expanded *ex vivo*, and transferred back to patients. Clinical benefit after ACT has been obtained in treatment of infection, various hematological malignancies, and some solid tumors; however, due to poor functionality and persistence of the transferred T cells, the efficacy of ACT in the treatment of most solid tumors is often marginal. Hence, much effort is undertaken to improve T cell function and persistence in ACT and significant progress is being made. Herein, we will review strategies to improve ACT success rates in the treatment of cancer and infection. We will deliberate on the most favorable phenotype for the tumor-specific T cells that are infused into patients and on how to obtain T cells bearing this phenotype by applying novel *ex vivo* culture methods. Moreover, we will discuss T cell function and persistence after transfer into patients and how these factors can be manipulated by means of providing costimulatory signals, cytokines, blocking antibodies to inhibitory molecules, and vaccination. Incorporation of these T cell stimulation strategies and combinations of the different treatment modalities are likely to improve clinical response rates further.

## Introduction

During the recent years, immunotherapy has emerged to be a powerful and potentially curative therapy for the treatment of various types of cancer and recurrent viral diseases. Adoptive cellular therapy (ACT) is a form of immunotherapy that involves the *ex vivo* isolation and expansion of antigen-specific T cells for adoptive transfer back to patients (1, 2). Although clinical benefit has been obtained in treatment of hematologic malignancies and melanoma, the efficacy of ACT in the treatment of most solid tumors is thus far limited since transferred T cells fail to function and persist *in vivo*. This is in sharp contrast to clinical results obtained with patients treated by ACT for virus-associated malignancies and recurrent viral infections. Here, sustained presence of functional virus-specific T cells is observed, even up to 9 years post-infusion (3–5). This prolonged presence of transferred virus-specific T cells translates into a high clinical response rate that is being observed in patients that are treated with these cells. The superior efficacy obtained with ACT in the treatment of viral infections and virus-associated malignancies compared to the treatment of most solid cancers can be attributed to several factors, including tolerance to tumor-associated antigens (TAAs) and inhibition of tumor-specific T cells due to the suppressive tumor environment. Moreover, also the necessity for extensive culturing of tumor-specific T cells to obtain sufficient numbers for infusion into patients greatly influences the quality of the T cells and, hence, persistence and anti-tumor efficacy *in vivo*. In addition to lessons that can be learned from studying T cells in a setting of viral infection, valuable lessons can also be learned by critical evaluation of results obtained with current protocols and, importantly, by improving our understanding of the underlying mechanisms. In this review, we will focus on current protocols of adoptive T cell therapy in cancer treatment, and discuss the various attempts to improve the clinical success rate of ACT by aiming to advance the quality of the infused T cells through delivery of costimulatory signals and cytokines, blocking of inhibitory signals and vaccination. As such, these developments are of interest for ACT improvement in cancer but also for other complicated T cell-dependent treatment modalities.

## Approaches of Act

One form of ACT involves expansion and infusion of natural T cells isolated from autologous tumors. Generation of tumor-infiltrating lymphocyte (TIL) cultures is performed by first culturing resected tumor fragments or tumor single-cell suspensions in medium containing IL-2 for 3–5 weeks followed by a rapid expansion protocol (REP) involving the activation of TILs using an anti-CD3 monoclonal antibody in the presence of irradiated peripheral blood mononuclear cells (PBMC) and IL-2 (2, 6, 7). Systemic administration of TILs to patients with advanced stage melanoma has mounted high and durable responses that resulted in objective clinical responses in >50% of the patients and complete regression in up to 24% of the patients (6, 8–10). However, such results have only been described for ACT in melanoma patients and not for other solid tumors. This is probably due to the high mutational load in melanoma giving rise to neoepitopes, which can serve as neoantigens facilitating tumor recognition by T cells (11–14). The stability of this neoantigen expression, however, is altering upon ACT demonstrating a dynamic interaction of the transferred T cells with their targets and advocates for ACT procedures inducing a broad tumor-specific T cell response (15).

In most medical centers, lymphodepletion before transfer is a standard part of the treatment (16). However, ~50% of the patients experience side effects of this pretreatment, which are mostly infection related, i.e., neutropenia and bacteremia (10). There is some evidence that alternative approaches can overcome the necessity to pre-condition the patient, e.g., selection of particular T cell clones, tailoring tumor-specific T cells to produce IL-12 or administration of low dose IFN-α (17–19). Another hurdle in ACT for solid tumors is the failure to successfully isolate TILs or expand TILs to sufficient numbers. In ACT for melanoma patients, these procedures are usually very efficacious, showing a success rate of more than 50% (7, 8). However, TILs harboring sufficient anti-tumor activity can rarely be generated from tumors other than melanoma and, moreover, for other types of cancer, adequate amounts of surgical/bioptic material is often not available (20).

One strategy to circumvent these limitations is genetic engineering of autologous T cells by lentiviral or retroviral transduction to express TCRs that recognize TAAs. Although a promising clinical response rate of 30% was observed in a clinical trial for melanoma patients using a high-affinity HLA-A0201-restricted MART-1 TCR, in 29 out of 36 patients severe off-target toxicity was seen in the skin, ears, and eyes as destruction of melanocytes also occurred at these sites (21). In a clinical trial where myeloma and melanoma patients were infused with autologous engineered T cells expressing an affinity-enhanced TCR against MAGE-A3, the first two patients died of cardiogenic shock. This severe cardiac toxicity was due to recognition of a MAGE-A3-unrelated protein expressed by normal cardiac tissue (22). This off-target activity is likely caused by the fact that an affinity-enhanced TCR was used instead of the low-affinity parental TCR against MAGE-A3. Thus, a major drawback of this approach is the (sometimes unidentified) expression of target antigens on healthy tissue resulting in unwanted cross-reactivity. Nevertheless, certain antigens, such as cancer-testis antigens (CTAs), do form an attractive target since they are expressed by a variety of tumor types, but usually not by adult tissue, with the exception of germline cells on which HLA class I and II is not expressed. In clinical trials, targeting of the CTA NY-ESO-1 antigen, 61% of synovial cell carcinoma patients and 55% of the melanoma patients demonstrated objective clinical responses without signs of off-target toxicity (23). Another report showed even an 80% response rate in multiple myeloma (24). Although TCR transduction allows the generation of tumor-specific T cells without the necessity to isolate TILs, a major limitation of this approach is the HLA-restriction. For example, transduction of a TCR that recognizes its antigen in the context of HLA-A*0201 is only functional in patients with the same HLA type.

An alternative approach to obtain T cells with anti-tumor reactivity without the complication of HLA-restriction is genetic engineering of T cells to express chimeric antigen receptors (CARs). CARs are constructed by linking an antigen-binding domain, usually a single-chain variable fragment (scFv), to an intracellular T cell signaling domain such as CD3-ζ (first generation CAR), and currently also including one or two costimulatory domains (second/third-generation CAR). The specific binding of CAR T cells occurs, thus, in a non-MHC-restricted fashion, yet antigen-binding results in T cell activation. The most impressive clinical results so far have been obtained using CAR T cells targeting CD19 in patients suffering from B cell malignancies (25–30). Mixing defined populations of CD4^+^ and CD8^+^ CAR T cells recognizing both CD19 further improved this therapy (31, 32). However, since all CD19-expressing cells are targeted using this approach, also non-malignant B cells are depleted. The drawback that healthy cells expressing the antigen are also targeted by CAR T cells has also been reported for CARs directed to her2/neu and carboxy-anhydrase-IX (33, 34). In addition to this on-target off-tumor effect, acute anaphylaxis and tumor lysis syndrome (TLS) occurs frequently after CAR T cell therapy, but most often observed is cytokine release syndrome (CRS) (35–37). It has been suggested that the incidence and severity of the CAR T cell-mediated toxicity is related to tumor burden and T cell infusion dose. To minimize toxicity in patients with a high tumor burden, treatment with a low T cell dose may be required (30, 32, 35, 37). Other strategies to overcome these adverse events include addition of a suicide gene (e.g., HSV-TK and *iCasp9*), whereby transferred cells can be selectively eliminated, and the generation of CAR T cells with dual specificity whereby each CAR targets a different tumor antigen and only engaging of both results in proper T cell activation and effector function (38–44).

Clinical trials using CAR T cells targeting other antigens than CD19 have, thus far, only shown limited anti-tumor efficacy. In a trial wherein neuroblastoma patients received CAR T cells recognizing the extracellular domain of L1-CAM, present on neuroblastoma cells, two out of six patients showed a clinical response (34). However, in two other trials, using CAR T cells specific for carboxy-anhydrase-IX and alpha-folate receptor to treat renal cell carcinoma and ovarian cancer, respectively, no clinical responses were observed (45, 46). The limited success for these CAR T cells may in part be due to antigen-independent CAR signaling due to clustering of CAR scFvs resulting in their early exhaustion. This tonic CAR signaling is observed for several CARs, except the CD19 CAR (47). Incorporation of the endodomain of 4-1BB (CD137), a costimulatory member of the TNF receptor (TNFR) superfamily, rather than a CD28 domain ameliorates this induction of exhaustion (47, 48). In addition, novel targets for CAR T cell therapy for solid tumors are on their way, which may have high clinical potential. For example, it was recently reported that CAR T cells can be engineered to target aberrantly glycosylated antigens on MUC1, which is expressed by multiple cancers, thereby providing a potential broad application (49).

Although CAR T cells and TCR transgenic T cells are favorable cancer treatment modalities, they usually target a single tumor antigen, which increases the chance of tumor escape (50, 51), and limits eradication of the often very heterogeneous tumors. The use of a combination of CARs with different antigen specificity or bispecific CARs could prevent antigen escape (52).

## Quality of Anti-Tumor T Cells

A major challenge in ACT is to obtain sufficient numbers of tumor-specific T cells for infusion into patients and, importantly, since durable clinical responses profoundly depend on persistence of the infused T cells, the transferred cells should have the capacity to persist long-term *in vivo* (53). Several reports suggest that the relative contribution to long-term persistence of T cells mainly comprises the least effector-differentiated memory T cells: central memory T cells (Tcm) and T memory stem cells (Tscm) (54). Tcm and Tscm circulate in the lymphoid organs and are endowed with an excellent expansion potential upon antigenic challenge as opposed to more differentiated memory T cells. Effector and effector memory T cells (Teff/Tem) home to tissues and respond to antigen with immediate effector function as compared to Tscm/Tcm, but have a reduced regenerative capacity (55). In addition, Tem in humans can be subdivided into cells that are either CD45RA^−^ or cells that re-express CD45RA^+^. The re-expressing cells, termed Temra, are thought to be the most differentiated memory cells, as these cells have low proliferative capacity, strong cytotoxic potential, and a higher susceptibility to apoptosis (56).

Tscm have the capacity to differentiate into Tcm and Tem, and display a superior potential to self-renew as evidenced by a positive correlation of the amount of infused Tscm with early expansion after transfer and absolute numbers of long-term persisting cells (57–59). However, very low numbers of Tscm are found in the periphery and extensive expansion would be required, which likely results in loss of memory potential (60, 61). The limitation of low natural frequencies can be bypassed by targeting the Wnt/β-catenin pathway in naive cells that results in arrested Teff differentiation and promotion of memory-like CD8^+^ T cells with Tscm features. Although targeting the Wnt signaling pathway appears to be an effective method to promote stemness and inhibit differentiation, this may restrict the proliferation and function; hence, further research is required for its suitability to improve ACT (62). An alternative method to generate sufficient Tscm is a procedure whereby human naive T cells are activated by CD3/CD28 engagement and culturing in the presence of IL-7, IL-15, and IL-21 (63, 64). Another approach currently being explored is based on inhibition of the Akt-signaling pathway during the *ex vivo* expansion of tumor-specific T cells resulting in the induction of early memory-like cells (65, 66). The advantage of this approach is that the *ex vivo* proliferation is not strongly inhibited and sufficient numbers of cells can be obtained for treatment. However, the role of Akt in T cell differentiation and metabolism needs to be further validated in order to determine if Akt inhibition could potentially be used in ACT protocols. Thus, although it is clear that Tscm have excellent stemness properties and much effort is being made to optimize isolation and expansion protocols, there are still some major hurdles and it is, therefore, not feasible yet to use these cells routinely for adoptive cell therapy.

A recent report demonstrates an alternative approach in which TCR transgenic CD8^+^ T cells were successfully reprogrammed into induced pluripotent stem (iPS) cells using a Sendai virus vector. After transfer into melanoma-bearing mice, iPS-derived T cells mediated potent anti-tumor activity. Nevertheless, their anti-tumor activity and persistence were comparable with their non-reprogrammed counterparts (67). Importantly, the Busch laboratory convincingly showed in mice that also Tcm have stemness and long-term persistence potential after transfer. Actually, both naive T cells and Tcm cells were highly efficient in inducing epitope-specific T cell populations during serial single cell adoptive transfers (68). Also, infused Tcm clones in monkeys and humans have shown to have the capacity to mount long-term persistent clonotypes, and furthermore CD19 CAR T cells derived from Tcm have superior anti-tumor effects (31, 59, 69, 70).

In the current point of view, both Tscm and Tcm seem to be *bona fide* T cell subsets to use in ACT. Moreover, also naive T cell subsets have the potential to establish long-term persistence allowing for prolonged anti-tumor activity (71, 72). However, these less-differentiated T cell subsets are not per definition superior in all tumor eradication settings. In cases of solid tumors where the level of tumor-antigen presentation by antigen-presenting cells in lymphoid organs is low, these T cell subsets may not be activated sufficiently to exit the lymphoid organs and invade the tumor to exert their anti-tumor effects. One strategy to overcome this hurdle is increasing the level of antigen presentation in the lymphoid organs by vaccination, which results in appropriate T cell stimulation (as will be discussed later). Another approach is co-infusion of Teff and Tem cells. These cells have direct effector function and have (extralymphoid) tissue migrating properties leading to tumor destruction (55, 73–75). Consequently, this may also lead to sufficient activation of the co-transferred Tcm/Tscm, which enables long-term anti-tumor immunity.

## *Ex Vivo* Expansion Protocols and Costimulation

The expansion protocols that are currently used to expand TILs or generate engineered tumor-specific T cells often mount expanded T cell pools with a highly differentiated phenotype that have lost CD28 expression, decreased expression of the costimulatory TNFR family member CD27 and more susceptibility to activation-induced cell death (AICD) (76–78). Approaches to obtain sufficient numbers of TILs with a favorable phenotype or to reprogram TILs or TCR engineered T cells to the preferred phenotype during *ex vivo* expansion include manipulation of critical costimulatory and cytokine signaling pathways. Costimulatory signals can be provided via agonistic antibodies and artificial APCs (aAPCs), of which the latter can either be cell-based or non-cell-based (79). An advantage of non-cell-based aAPCs over cell-based aAPCs is that they can be engineered to be magnetic, which makes removal of the cells before infusion of the T cell product straightforward. Also bio-degradable particles can be designed of which removal is not necessary. One of the costimulatory pathways known to be critical for priming T cells, the CD28 pathway, is currently implicated in ACT protocols for *ex vivo* expansion and transduction. Another candidate is 4-1BB, which is expressed on activated T cells and upon triggering enhances T cell responses by promoting proliferation, survival, and effector function, and by regulating the suppressive potential of regulatory T cells (Tregs) (80). Comparison of aAPCs providing costimulation via CD28 or 4-1BB showed that signaling through 4-1BB preferably expands memory CD8^+^ T cells, whereas CD28 costimulation favors expansion of naive cells. In addition, the CD8^+^ T cells that received 4-1BB signals displayed improved cytolytic function (81). Interestingly, enhanced 4-1BB costimulation through an agonistic antibody has been shown to rescue expression of CD27 and CD28 on post-REP CD8^+^ TILs, improved expansion of CD8^+^ T cells, and increased responsiveness to antigenic re-stimulation and increased expression of the CD127 (IL-7Rα) (82, 83). Also, when combined with a potent TCR trigger, signaling through 4-1BB induces prominent upregulation of CD25 (IL-2Rα) and IL-2 (84). Thus, while generating tumor-specific T cell pools from naive cells, 4-1BB triggering could promote the generation of T cells capable of expanding upon secondary challenge. Another costimulatory molecule that could potentiate *ex vivo* culturing of tumor-specific T cells is CD27. The interaction of CD27 with its ligand CD70 has been shown to be important for IL-2-mediated T cell activation and *in vitro* activation of human T cells with anti-CD3 in the presence of an agonistic CD27 antibody showed comparable expansion potential as stimulation through 4-1BB (85). On the other hand, *in vitro* experiments have shown that in a co-culture of naive CD4^+^ T cells with CD70 expressing tumor cells, Tregs accumulate because of increased IL-2 production by non-Treg CD4^+^ cells (86). Other costimulatory members of the TNFR superfamily include OX40 (CD134), HVEM, and GITR, and agonistic antibodies targeting these molecules could also potentially be used to improve REP cultures. OX40 has been described to promote T cell expansion and survival, the latter probably by regulating BCL-2 and BCL-x_L_ expression (80, 87, 88). It has been shown that ligation of OX40 increases expression of IL-7Rα on antigen-specific CD8^+^ T cells, which leads to enhanced survival and accumulation upon IL-7 signaling, and combining OX40 and 4-1BB costimulation further enhanced this effect (89). Thus, to further improve the *ex vivo* culturing procedure, targeting of two or more costimulatory pathways simultaneously can be taken into consideration. Importantly, although the signal strength that is delivered to the T cells should be robust enough for proliferation, it should not result in an overall terminal differentiation of the T cells. An alternative approach is to make combinations of an agonistic antibody with cytokines that prevent overt differentiation, as will be discussed hereafter. To be able to select the most favorable agonist–cytokine combinations, it would be highly recommendable to expand our knowledge regarding the effect on the expression of cytokines and cytokine receptors by targeting the costimulatory pathways simultaneously.

## *Ex Vivo* Expansion and Cytokines

An alternate strategy to boost cultured T cells and modulate the phenotype is via cytokine-mediated signals. The common-gamma chain (γ_c_)-cytokine IL-2 is long been known to massively expand T cells, and high doses of IL-2 have been used to establish and expand ACT T cell cultures for more than 20 years (90). Enforced expression of IL-2 by the T cells themselves results also in prolonged survival *in vitro* and maintains the tumor specificity and function (91, 92). However, IL-2 can promote differentiation of T cells (93, 94), which may lead to an unfavorable phenotype for ACT usage. So strategies to optimize *ex vivo* T cell cultures for ACT involving the (co-)use of alternative cytokines are fully explored. Next to IL-2, other γ_c_-cytokines, such as IL-7, IL-15, and IL-21, have been described to play a role in memory T cell formation, proliferation, and survival, yet result in a lower degree of T cell differentiation but are still able to enhance anti-tumor responses (95–99). Also IL-12 and IFN-α, non-γ_c_-cytokines, hold promise to enhance the efficacy of ACT. IL-12 has been shown to play an essential role in T cell differentiation and memory formation and IFN-α is important in driving memory cell development (100–102). Use of these cytokines in *ex vivo* T cell cultures present a promising moiety to yield T cells with an improved capacity to respond (103, 104). T cells forced to secrete IL-12 benefited also of this cytokine during culture (105).

In particular combinations of cytokines have shown encouraging results. In expansion, protocols using naive T cells as a starting source, different combinations of IL-2, IL-7, IL-15, and IL-21 have proven to efficiently expand T cells and result in populations expressing early-differentiation markers, such as CD27, CD28, CD62L, and CD127 (64, 106–108). It has also been shown that CAR T cells can be efficiently expanded using a protocol devoid of IL-2, using CD3 and CD28 stimulation in the presence of IL-7 and IL-15 (109). Interestingly, also in TIL cultures and TCR/CAR engineered T cell cultures that are established in the presence of anti-CD3 and IL-2 and usually display a substantial degree of differentiation, cytokine cocktails were able to establish T cell populations with a less-differentiated phenotype (76, 110, 111). This suggests that cytokine cocktails can be used to reprogram late differentiated T cells. Besides delivery of cytokine-mediated signals via cytokine supplementation to the cultures, aAPCs can be designed to express cytokines or T cells can be triggered or engineered to produce abundant cytokines themselves (99, 112–114). The advantage of aAPCs is that they can be designed to simultaneously provide costimulation and cytokine-mediated signals (112, 115). Irrespective of the cytokine delivery manner, more research is required to pinpoint the amount, ideal timing, and combination of cytokines for *ex vivo* cultures (108, 110). Moreover, requirements for establishing cultures from naive T cells or previously primed T cells are likely to be different. For example, addition of IL-21 causes naive T cells to significantly expand, while memory T cells fail to do so (98). Nevertheless, in both subsets, IL-21 signaling increases CD28 expression.

## *In Vivo* Costimulation

Another approach to improve ACT is by enhancing the T cell expansion and function after transfer. The essentiality of costimulatory pathways has been demonstrated in experimental settings of adoptive transfers showing, e.g., that CD27- and CD28-mediated costimulatory signals are important for expansion of both naive and memory CD8^+^ T cells upon transfer (116, 117). To study the benefit of augmenting costimulatory pathways in patients after T cell infusion, preclinical studies and clinical trials have been performed exploring the use of agonistic antibodies against TNFR superfamily members 4-1BB, OX40, GITR, CD27, HVEM, and CD40. The promise of these molecules in cancer immunotherapy has been reviewed recently (118–120).

Besides expansion, an additional beneficial effect delivered by costimulation is induction of T cells with the capacity to produce IL-2 that acts in an autocrine manner (84, 121). In contrast to exogenous IL-2, either provided *in vitro* or *in vivo*, autocrine IL-2 seems to be highly beneficial for both the (secondary) expansion potential and survival of CD8 T cells (113, 122). However, in order to be able to optimize ACT protocols further and minimize the chance of severe adverse side effects as observed in the Phase I clinical trial with anti-CD28 and to a lesser extent in the Phase II study with the anti-4-1BB antibody Urelumab (NCT00612664), which was associated with a high incidence of severe hepatitis, a better understanding of the underlying mechanisms by which these antibodies exert their effects is crucial (123, 124). In preclinical models, agonistic 4-1BB contributes to tumor regression by promoting survival and avoiding AICD of CD8^+^ T cells and more importantly in the context of this review; in models using OVA-expressing tumors, it has been demonstrated that a combination of agonistic 4-1BB antibody and transfer of OVA-specific CD8^+^ T cells significantly improves tumor control (125, 126). Whether combining ACT and 4-1BB agonists enhances anti-tumor activity in humans has not yet been assessed, but when used as a monotherapy, 4-1BB antibodies seem to have some anti-tumor activity. Although two 4-1BB agonists have already been used in clinical trials, only recently more insight into the mechanisms by which anti-tumor effect is exerted, is obtained, and it has become clear that at least in preclinical models systemic 4-1BB activation induces a phenotype of CD4^+^ and CD8^+^ T cells that is characterized by high expression of the T-box transcription factor Eomes, KLRG1^+^, and high cytotoxic capacity (125, 127–129). KLRG1 marks Tem and Teff cells and as already mentioned above, ACT of Tem and Teff cells combined with less-differentiated cells might be beneficial. Furthermore, agonistic 4-1BB antibody treatment correlated with decreased expression of the inhibitory receptors programmed death-1 (PD-1) and Lag3.

OX40 signaling can enhance T cell differentiation and survival via effects on IL-2 and IL-7-mediated signaling, and via increasing the anti-apoptotic molecules Bcl-2 and Bcl-xL (130) Essentially, providing OX40 triggering augmented anti-tumor activity in a preclinical model of adoptive T cell transfer mediated by both CD4^+^ and CD8^+^ T cells (131). Conflicting results are reported on whether Treg responses are inhibited or promoted by OX40, which is most likely due to differences in dose and/or timing of OX40 ligation, and may depend on the model/setting (132). Although unraveling the precise mechanism of OX40 agonists remains a challenge, anti-OX40 has already been used in a Phase I clinical trial for patients with metastatic solid malignancies, albeit not in ACT settings (133). Results were promising, and indicated enhanced proliferation of CD4^+^ and CD8^+^ T cells that coincided with regression of at least one metastatic lesion in 12 out of 30 patients. Tregs in the tumor showed a higher expression of OX40 compared to peripheral blood Tregs.

Explored as well, albeit to a lesser extent are agonistic CD27 antibodies (134, 135). Promising results were reported in preclinical models, and are likely related to improved CD27-mediated T cell expansion, survival, and function (77, 136–138). Conversely, it has also been reported that CD27 signaling can increase survival of Tregs *in vivo* and thereby promote tumor progression (86). The Teff:Treg ratio in the tumor has been suggested to determine whether CD27 agonist will promote or diminish tumor control (139).

An indirect way to improve the efficacy of transferred T cells is via administration of agonistic antibodies to CD40 resulting in activation of APCs, such as dendritic cells (DCs) (120). Consequent upregulation of costimulatory molecules on the APCs then provide the necessary stimulatory signals to activate tumor-specific T cells. In addition to DCs, CD40 antibodies also activate other myeloid cells (140) and the activity can also depend on complement-mediated cytotoxicity (CMC) or antibody-dependent cell-mediated cytotoxicity (ADCC), or even be immune effector independent when CD40 is expressed on tumor cells (141–146). Important to note is that CD40 triggering in malignant cells is able to promote tumor cell proliferation leading to tumor progression (147, 148). Likely depending on one or more of the above-described mechanisms, targeting of CD40 has already been proven a promising strategy in several preclinical models and clinical trials against solid cancer (120, 149–154). Also in preclinical models of ACT, agonistic CD40 antibodies promote tumor-specific T cell expansion and enhanced anti-tumor activity (155, 156). Thus, clinical effectiveness in ACT has potential given that CD40 antibody-associated toxicity is managed (152, 157). So far three agonistic CD40 antibodies, which differ in their agonistic activity, have been tested in clinical trials. The strongest agonistic antibody, CP-870.893, is a humanized antibody of an IgG2 isotype. Human IgG2 antibodies typically interact marginally with Fc receptors and are, therefore, not very effective mediators of CMC and ADCC (158). Nevertheless, this antibody is a potent activator of macrophages and DCs and can thereby mediate T cell-dependent anti-tumor immune responses, which suggests that it has the potential to enhance ACT. The two other antibodies that have been tested in the clinic (i.e., Dacetuzumab and ChiLob 7/4), displaying less agonistic activity compared to CP-870.89, are of an IgG1 isotype and, hence, are more potent mediators of CMC and ADCC, making them less suitable for combinations with T cell transfer (120). Adverse effects that were observed after CD40 antibody treatment include CRS and liver damage (120). Targeting of CD40 is also possible by imposed expression of CD40L (CD154) on the transferred T cells. In an experimental model, CD19-specific CAR/CD40L T cells displayed increased cytotoxicity and enhanced tumor eradication (159).

Also currently under investigation in clinical trials is an agonistic antibody against GITR. In multiple animal models of cancer, this antibody has proven to exert anti-tumor immune responses by providing costimulatory signals to T cells and skewing the balance between induced Treg and T_H_9 cell differentiation in favor of T_H_9 (160–162). In a preclinical adoptive transfer setting, agonistic GITR antibody has shown to increase the polyfunctionality of the transferred T cells and reduce the frequency of Tregs in the tumor, resulting in tumor regression (163). Repetitive doses of a GITR agonist is, however, potentially toxic (164).

Taken together, considerable progress has been made in dissecting the mechanisms by which agonistic antibodies to costimulatory molecules exert their anti-tumor effects but further unraveling is required to be able to implement this therapy into patients receiving ACT. Importantly, in case of treatment with such powerful agonists, also the mechanisms underlying the adverse immune-mediated side effects require attention. Undoubtedly, the effects of agonistic antibody administration are often multifaceted thereby making it challenging to predict treatment outcome. Attempts to minimize the chance of antibody-induced toxicity could include pretreatment with corticosteroids and local administration of agonistic antibodies (151, 152, 154).

As mentioned before, second-generation CAR T cells contain a costimulatory domain placed in series with CD3ζ and thereby costimulation is provided per definition upon target recognition. Since it is well appreciated that T cells require costimulation for proper activation, it is not surprising that incorporation of costimulatory domains advanced CAR T cell treatment. Alike for providing costimulation by agonistic antibodies, the choice of the costimulatory signaling domain influences CAR T cell functionality and persistence, i.e., by differential regulation of down-stream signaling expression. Most extensively explored are CAR T cells with incorporated CD28 and 4-1BB signaling domains and although treatment with both CAR T cells have resulted in clear clinical responses, comparisons showed prolonged persistence and ameliorated exhaustion of CAR T cells using the 4-1BB domain (47, 165, 166). Alternative signaling domains that have been integrated include domains of CD27, ICOS, and OX40 (165, 167–169). In third-generation CAR T cells, attempts to further enhance anti-tumor activity and long-term persistence rely on incorporation of two costimulatory domains. So far, combinations of CD28 with 4-1BB and CD28 with OX40 have shown to be promising, resulting in T cells having potent effector functions and improved capacity to persist long term (168, 170). In a small pilot trial, a CD20-specific CAR with CD28 and 4-1BB costimulatory domains has been tested in four relapsed indolent B cell and mantle cell lymphoma patients and the data suggest improved CAR T cell persistence (171).

In addition to improving T cell function by triggering costimulatory pathways, inhibitory pathways can be blocked and this strategy, also known as immune checkpoint blockade, has led to significant clinical advances in cancer immunotherapy (172–174). Several reports show that combinations of ACT and blockade of inhibitory molecules, such as CTL-associated antigen 4 (CTLA-4) and PD-1, have the potency to augment anti-tumor efficacy and increase T cell persistence (175–179). An alternative method in which PD-1-mediated inhibition was turned into CD28-mediated costimulation by generating PD-1–CD28 fusion receptors was also effective in ACT (180). Nevertheless, although targeting of either costimulatory or inhibitory pathways for the benefit of ACT may improve anti-tumor responses, for achieving greater clinical response rates, combinations of the two might be required and are currently under investigation. So far this approach has yielded encouraging results as evidenced by inhibition of tumor growth in preclinical settings, including ACT cancer models (181–185). Decreased tumor progression coincided with an increase in Teffs and a decrease in Tregs and myeloid suppressor cells at tumor sites. This shift from a more immunosuppressive to a more immunostimulatory tumor environment might explain the potent effects of these antibody combinations.

## Cytokines *In Vivo*

Adoptive cellular therapy using TILs generally includes *in vivo* administration of high-dose IL-2 to improve proliferation and survival of the transferred TILs. Unfortunately, exogenous IL-2 treatment has two major drawbacks; it is often associated with severe toxicity and can promote Treg proliferation. It has been reported that the number of doses of IL-2 that are administered after adoptive TIL transfer is positively correlated with Treg reconstitution after lymphodepletion and, furthermore, that the degree of Treg reconstitution is inversely correlated with the patient’s response to treatment (186).

Attempts to circumvent IL-2-induced toxicity and Treg stimulation have been made. A straightforward measurement is by reduction of the IL-2 administration (187). Tailoring CD8^+^ T cells to augmented autocrine IL-2 production seems an alternative promising manner, which increases the availability of IL-2 to the right cell without promoting Treg proliferation. This can be achieved using retroviral or lentiviral transduction and this would especially be feasible in situations where transduction is already required. For instance, in case of generating TCR engineered T cells, but in fact also TILs can be transduced in the same manner. Recently, we have shown in a preclinical model that CD8^+^ T cells cultured in the presence of IL-7 and IL-15 that are forced to overexpress IL-2 display improved persistence and expansion potential after transfer and subsequent vaccination (113). Consequently, this heightened anti-viral and anti-tumor immunity *in vivo* compared to mock transduced cells. Notably, after *in vivo* secondary challenge, the cells with elevated autocrine IL-2 efficiently re-expanded yet also expressed IL-7Rα, suggesting that although these cells underwent prolonged IL-2 signaling, they still seem to be of a less-differentiated phenotype, which may be related to the transduction procedure in the presence of IL-7 and IL-15. In addition, we did not observe any alterations in Treg homeostasis (113). ACT with human T cells overexpressing IL-2 has also been explored yielding promising results with respect to longevity but large clinical studies should be performed to determine if IL-2 over-expressing T cells result in clinical benefit (44, 92). Likely, ACT approaches with IL-2^+^ T cells are most successful when they are combined with vaccination given the prominent role of autocrine IL-2 production for secondary expansion of CD8^+^ T cells (113, 122). As discussed before, another strategy to circumvent exogenous IL-2 administration is to provide agonistic antibodies to costimulatory receptors that promote autocrine IL-2 production in T cells.

Other cytokines than IL-2 have also been explored to enhance ACT-mediated T cell responses. IL-7 and IL-15 are crucial cytokines for lymphoid homeostasis by playing an important role in orchestrating the survival of naive and memory T cells and memory cell differentiation (188, 189). Increased availability of IL-7 and IL-15 has been shown to be an important mechanism by which a lymphodepleting regimen improves the engraftment of the adoptively transferred T cells and, hence, the success of ACT (190–192). Preclinical ACT models, in which the effect of exogenous IL-7 and IL-15 on tumor outgrowth has been tested, demonstrated that both cytokines can improve tumor control, including in vaccinated lymphodepleted or immunodeficient hosts (193–195). Recently, a phase I clinical trial has been conducted to determine safety, adverse event profiles, and the maximum tolerated dose of rhIL-15 in humans (196). Patients with metastatic melanoma and metastatic renal cancer were infused with different doses of IL-15 (0.3/1.0/3.0 μg/kg/day) for 12 consecutive days and this treatment regimen resulted in markedly altered homeostasis of mainly NK cells, γδ cells, and to somewhat lesser extent of memory CD8^+^ T cells. No clinical responses according to the RECIST criteria (197), which includes the persistence of the cells after transfer, were observed and the maximum tolerated dose was determined to be the lowest used dose. Because of clinical toxicity caused by strong cytokine production, the authors stated that rhIL-15 is too difficult to administer intravenously and suggest developing alternative dosing strategies and new trials to assess this are being conducted (196). IL-7 administration is tolerated better in humans, but anti-tumor efficacy requires further evaluation (198, 199).

As for IL-2, systemic IL-15-mediated toxicity might be circumvented by tailoring tumor-specific T cells to express IL-15. In that way, the IL-15-mediated effects are likely confined to the tumor environment, eluding systemic toxicity. In experimental models, it was shown that IL-15-expressing CD8^+^ T cells improve anti-tumor activity (95), and human IL-15 secreting cells perform also well *in vivo* (43, 200, 201).

The more recently discovered member of the γc cytokine family, IL-21, has also been explored as an anti-cancer treatment and IL-21 monotherapy of thymoma and melanoma in mice has shown to result in improved CD8^+^ T cell-mediated anti-tumor responses with augmented long-term survival (193, 202–204). IL-21 treatment prolonged persistence of endogenous and adoptively transferred tumor-specific transgenic CD8^+^ T cells, which was mainly attributed to IL-21-mediated promotion of survival (202). Additionally, it has been shown that IL-21 is able to potentiate tumor-specific antibody responses, which resulted in complement-mediated tumor cell lysis (204). Combination of cytokines involving IL-21 demonstrated further enhancement of anti-tumor immunity compared to IL-21 as a monotherapy. In a study wherein mice were challenged with B16F10 melanoma, treatment by adoptive transfer of transgenic tumor-specific CD8^+^ T cells, combined administration of IL-21 and IL-2 and vaccination resulted in higher absolute numbers of circulating tumor-specific T cells and improved tumor-free survival compared to therapy with IL-2 or IL-21 alone (203). In addition, in a model using murine B16 melanoma cells that were transfected to secrete IL-21, it was shown that local presence of IL-21 can also promote anti-tumor immunity by preventing IL-2-mediated Treg induction (205). Experiments in the B16 model has shown that mixing IL-21 with IL-15 improves expansion of transferred tumor-antigen-specific CD8^+^ T cells and enhances tumor control after vaccination (106). Clinical trials using IL-21 as a single agent in melanoma and renal cell carcinoma show that this cytokine is well tolerated and favorable clinical responses have been observed as evidenced by patients in which disease stabilized (206, 207). To our knowledge, the anti-tumor effect of IL-21 in human ACT has not been addressed yet. A study by Markley and Sadelain showed that forced expression of IL-7 and IL-21 by CD8^+^ T cells resulted in improved rejection of systemic lymphoma compared to T cells that overexpressed IL-2 or IL-15 (99). However, in these experiments, vaccination was not provided post-transfer, which may have resulted in improved expansion of the transferred IL-2^+^ cells.

Anti-tumor efficacy of some cytokines that do not belong to the γc cytokine family has been explored as well. In preclinical models, administration of IL-12 caused tumor regression and promoted survival of tumor-bearing animals. This provided the rationale for applying IL-12 treatment in a clinical setting, however, translation into the clinic was hindered by severe toxicity (208, 209). To be able to explore IL-12 as a treatment modality, multiple attempts have been made to design a safe IL-12-based treatment, including different administration schedules and routes and intratumoral delivery (210). Several reports explored the therapeutic efficacy of tumor-specific T cells designed to express IL-12. In experimental settings, T cells modified to produce IL-12 improved tumor eradication (19, 101, 211–213). Promising results were obtained using IL-12 gene transduced human TILs. Here, different doses of IL-12-producing TILs were infused into metastatic melanoma patients and higher doses resulted in better clinical responses (214). Nevertheless, IL-12 transduced TILs did not persist for more than a month. In addition, higher cell doses led to severe adverse side effects attributable to the secreted IL-12 (214). The lack of persistence of the transferred T cells that is observed might be due to a negative feedback loop that involves IL-10 production to limit ongoing T cell activation (215). So far, these reports suggest that ACT with IL-12-producing T cells has potential but improving the longevity of the cells and measures to prevent IL-12-mediated toxicity are vital for further application.

IFN-α is known for its direct anti-tumor effect and is currently a frequently used cytokine for the treatment of cancer. It has also been recognized that IFN-α promotes T cell activation, survival, expansion, and memory formation through activation and differentiation of DCs (216). The mechanism by which IFN-α mediates memory formation is suggested to be mediated by enhancement of IL-15 presentation of DCs to T cells (217). Moreover, IFN-α can also affect T cell expansion directly. This so-called signal 3 further amplifies the signals T cells receive via the TCR (signal 1) and costimulatory receptors (signal 2) (218–220). However, due to severe adverse events caused by IFN-α administration, treatment is discontinued in up to 50% of the cases. Frequently observed symptoms of IFN-α-related toxicities include “flulike” symptoms, fatique, anorexia, and nausea (18, 221). To improve tolerability of IFN-α treatment, a polyethylene glycol (peg) moiety was added, resulting in a longer half-life. This allowed for less-frequent administration and, hence, less toxicity. Accordingly, discontinuation rates of IFN-α treatment were lower when a pegylated form was used (up to ~25%). To further enhance the anti-tumor effect of cytokines in ACT, blocking antibodies to inhibitory receptors can be co-administered, e.g., IL-21 and CTLA-4 (222).

Overall, we conclude that although combining ACT with cytokine treatment seems a promising approach, as hold true for treatments that trigger costimulatory pathways, great care must be taken in applying cytokine therapy. The immune effects are not solely confined to the infused T cells and affect many other cells, frequently leading to severe systemic toxicities.

## Vaccination Provided Post Transfer

Therapeutic cancer vaccines have the potential to mediate clinical benefit, even as a monotherapy, providing the rational to consider it as an approach to improve ACT (223). Preclinical tumor models provided the insight that vaccination can improve ACT and strategies that have been used to aid anti-tumor efficacy in ACT include vaccination with viruses encoding tumor antigens, long peptides, peptide-pulsed DCs, and DNA vaccination (224–229). Vaccination predominantly seems to improve anti-tumor responses by enhancing tumor infiltration, persistence, and IFN-γ production of adoptively transferred T cells. Also in clinical trials, the potential of vaccination to enhance ACT has been explored, but thus far clinical success is marginal (230–233). An encouraging approach by Rapoport and colleagues showed that in the setting of autologous HSC transplantation for multiple myeloma pre-transplant vaccination, adoptive transfer of *in vivo* vaccine-primed T cells and subsequent vaccinations led to significant improvement of immunity in patients that would otherwise suffer from severe immunodeficiency due to high-dose chemotherapy (233). Since then, similar strategies have been applied in anti-cancer treatment in patients; autologous vaccine-primed lymphocytes were expanded *ex vivo* and adoptively transferred accompanied by vaccinations. Using this strategy, promising results have been obtained with respect to enhancement of the tumor-specific T cell response, but clinical activity remains to be further validated (234–237). More recently, two preclinical reports showed enhancement of CAR T cell-mediated anti-tumor responses by vaccination. Both studies were conducted using bispecific T cells targeting CMV and CD19, and vaccination consisted of the CMV peptide pp65 presented by either T cells or CD40L and OX40L expressing K562 cells (238, 239). Compared to mice receiving vaccination with an irrelevant peptide, control of tumor cell growth was improved and this coincided with increased frequencies of CAR–CMV–CTLs, suggesting that CMVpp65 stimulation expanded the bispecific T cells efficiently.

Thus vaccination as a modality to enhance ACT has so far not been explored in great detail and clinical trials using this approach have so far not yielded outstanding results. One of the reasons for this might be that the responsiveness of the adoptively transferred T cells to the vaccine is poor. As aforementioned pointed out, vaccination may be best suitable for less-differentiated T cells producing IL-2. Another possible explanation is that the immunosuppressive tumor environment is hampering T cell activation (240). Strategies to improve vaccination in the context of ACT include combination with peritumoral administration of TLR ligands and TLR-based adjuvants (226, 241).

Additional of great importance is the antigen that is used for vaccination. Often TAAs are used for vaccination. The advantage of this approach is that it is broadly applicable as it allows treatment of most patients with a certain tumor type. However, the specific T cell response toward the TAA can be blunted by central tolerance mechanisms. By contrast, T cells reacting to neoantigens expressed by tumors are not centrally tolerized (240). However, these antigens harbor unique mutations in a patient and, thus, targeting these antigens would require the production of personalized vaccines. This is a topic of intense interest and future studies should resolve the feasibility of such approaches.

Taken together, it is clear that strategies to improve ACT by vaccination need to be optimized and it seems that vaccination as a single modality to enhance this treatment is not sufficient.

## Concluding Remarks

**Figure 1:**
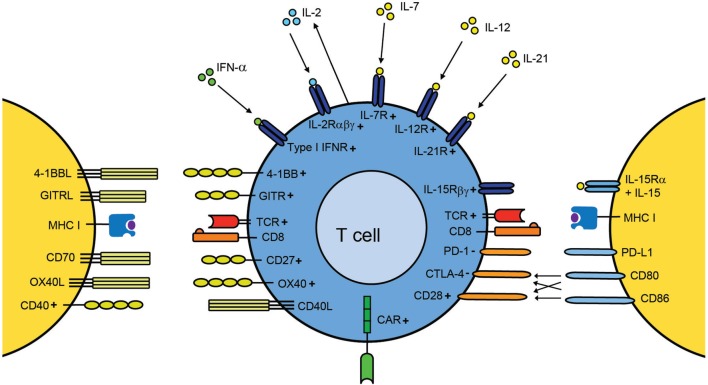
**A T cell centric view of improving adoptive T cell therapy by provision of T cell stimulation signals**. Upon transfer of *ex vivo*-expanded disease-specific T cells into the host, these cells recognize their cognate antigen in the context of MHC molecules via their TCR. Amplification of costimulatory signals (e.g., via agonistic antibodies) can be used to additionally stimulate T cells. Antibodies against CD28, CD27, 4-1BB, OX40, and GITR have been evaluated in preclinical models and clinical trials for their capacity to enhance T cell function. Targeting of costimulatory receptors can also be used during the *ex vivo* expansion of the T cells. In case of CAR T cells, recognition occurs via the CAR, a chimeric TCR that already provides a certain degree of costimulation. Blockade of inhibitory molecules, such as CTLA-4 and PD-1 by antibodies after transfer, counteracts suppressed T cells thereby improving T cell activity. In addition, inflammatory cytokines are able to provide signals for enhancing expansion, differentiation, and migration. Cytokines, such as IFN-α, IL-2, IL-7, IL-12, IL-15, and IL-21, have shown to have the capacity to enhance T cell efficacy either during *ex vivo* culturing or after adoptive transfer. Autocrine production of IL-2 is a vital property for secondary population expansion, and enhancing autocrine IL-2 is a promising way to improve T cell therapies. The + and − symbols indicate positive and negative signaling, respectively.

Currently multiple clinically approved immunostimulatory antibodies and cytokines are available that target a multitude of receptors expressed by T cells (Figure 1). It is expected that the agents targeting these receptors as well as the number of receptors that are targeted will increase in the coming years. The anti-tumor activity and persistence of infused T cells is highly dependent on the costimulatory pathways that are triggered after T cell transfer and on the expressed cytokine receptors. Unfortunately, the question as to which of the many T cell-stimulating pathways need to be activated during ACT to attain T cells that exert a superior anti-tumor effect and are able to persist long-term has no unanimous answer. *Ex vivo* culture methods should be designed in such way that the expression of the appropriate receptors on particular T cell subsets is induced, and this holds true for (autocrine) cytokine production as well. To predict the expression pattern, a detailed understanding of the regulation of these receptors is essential. Once this question has been addressed, the next challenge would be to make sure that the transferred T cells remain functional longitudinally, which involves likely a certain degree of heterogeneity of T cell subsets expressing various costimulatory and cytokine receptors.

Moreover, it is important to keep in mind that the effect of delivery of antibodies or cytokines to patients in order to improve survival, accumulation, and anti-tumor efficacy of the transferred T cells is not confined to the transferred T cells alone, but can affect also host cells bearing the appropriate receptor, potentially resulting in severe toxicity. Due to these toxicity issues, the overall results of using cytokines and agonistic antibodies against immune costimulators may have been modest with respect to the anti-tumor activities in clinical trials. It is conceivable that also by enhancing the quality of engineered T cells (including CAR T cells), which generally already recognize their targets with good affinity, the provision of (additional) costimulation or cytokines might potentiate cross-reactivity or toxicity. An approach to circumvent treatment-related adverse effects includes local administration or specific targeting to the tumor site (152, 226, 242). Furthermore, combinations of treatment modalities are likely to reduce the dose that is required for clinical responses and this might avoid severe adverse effects. On the other hand, certain combinations, even at lower concentrations might result in unexpected toxicity. In addition to strategies to enhance ACT that have been discussed in this review, i.e., by providing costimulation, blocking inhibitory molecules, cytokines, and/or vaccination, the T cell quality can be further enhanced by changing the tumor microenvironment to induce a more favorable milieu for T cells. Those strategies, which are beyond the scope of this review to be discussed in detail, include therapies counteracting myeloid-derived suppressor cells (MDSCs), neutralization of tumor acidity, chemotherapy, inhibition of IDO, and treatment with antibodies against immune suppressive cytokines (243–248). Finally, as discussed vaccination provided post transfer as an approach to enhance the efficiency of ACT is promising, yet such a combined treatment requires substantial effort to make it clinically successful.

Taken together, ACT holds its promise as an effective anti-cancer treatment but improvement is required. Concluding from the aforementioned discussion, the inclusion of T cell costimulation and cytokines should be an integral part for optimization of ACT protocols. In addition, combinations with other immune therapies, such as vaccination are expected to further improve the clinical success rates of ACT.

## Author Contributions

AR and RA contributed equally to the writing of the manuscript.

## Conflict of Interest Statement

The authors declare that the research was conducted in the absence of any commercial or financial relationships that could be construed as a potential conflict of interest.
